# How the COVID-19 crisis affected diverse older adults: A mixed methods case series

**DOI:** 10.1093/geroni/igab046.1659

**Published:** 2021-12-17

**Authors:** Ladda Thiamwong

**Affiliations:** College of Nursing, University of Central Florida, Orlando, Florida, United States

## Abstract

The coronavirus disease (COVID-19) pandemic has magnified inevitable physical, mental and social health consequences, especially in Hispanic older adults who experience health disparities and ageism. Even though physical distancing has been adopted as a key strategy to help reduce further spread of COVID-19, prolonged periods of physical distancing may worsen existing health problems. This study aims to explore how the COVID crisis affected diverse older adults. An explanatory sequential mixed methods design was utilized. Quantitative data were collected by questionnaires via Qualtrics survey and qualitative data were collected by individual phone interviews with four open-end questions. One in 4 older adults lives alone and one in 20 has no friend to call on. More than half of the participants were afraid of COVID and a fourth of them were afraid of losing their life to COVID. Participants identified keeping themselves busy as key to staying healthy during the pandemic.

